# Data on antigen recognition hindrance by antibodies covalently immobilized to Protein G magnetic beads by dimethyl pimelimidate (DMP) cross-linking

**DOI:** 10.1016/j.dib.2018.12.057

**Published:** 2018-12-21

**Authors:** Marcelle A. Caminha, Virginia Maria B. de Lorena, Wilson de Oliveira Júnior, Jonas Perales, Paulo C. Carvalho, Diogo B. Lima, Maria da Glória A.M. Cavalcanti, Sílvia M. Martins, Richard H. Valente, Rubem F.S. Menna-Barreto

**Affiliations:** a Laboratório de Biologia Celular, IOC, Fiocruz, Rio de Janeiro, RJ, Brazil; b Laboratório de Toxinologia, IOC, Fiocruz, Rio de Janeiro, RJ, Brazil; c Laboratório de Imunoparasitologia, CPqAM, Fiocruz, Recife, PE, Brazil; d PROCAPE, UPE, Recife, PE, Brazil; e Computational Mass Spectrometry & Proteomics Group, ICC, Fiocruz, Curitiba, PR, Brazil; f Mass Spectrometry for Biology Unit, CNRS USR 2000, Institut Pasteur, Paris, France

## Abstract

The data presented herein is related to the article entitled "*Trypanosoma cruzi* immunoproteome: calpain-like CAP5.5 differentially detected throughout distinct stages of human Chagas disease cardiomyopathy" [1]. Electrophoretic analyses under denaturing and reducing conditions indicate that covalent immobilization of human IgG to Protein G magnetic beads by cross-linking with 50 mM dimethyl pimelimidate hinders the recognition of *T. cruzi* antigens in immunoprecipitation assays.

**Specifications table**

**Table:** 

Subject area	Biology
More specific subject area	Immunoproteomics
Type of data	Figures
How data was acquired	SDS-PAGE under denaturing and reducing conditions (Laemmli). Image files (TIFF) of gels were acquired using ImageScanner III (GE Healthcare) and Adobe Photoshop Elements (Adobe Systems Inc.)
Data format	Formatted images
Experimental factors	IgG pools from human serum sample was initially purified to > 95% homogeneity (SDS-PAGE + shotgun mass spectrometry)
Experimental features	Purified human IgG pool was covalently bound to Protein G magnetic beads prior to immunoprecipitation attempts of *Trypanosoma cruzi* immunoreactive proteins.
Data source location	Human serum was collected at the "Ambulatório de Doença de Chagas e Insuficiência Cardíaca" in Recife, Brazil, the bloodstream form of *T. cruzi* and the experimental data were obtained at the Instituto Oswaldo Cruz in Rio de Janeiro, Brazil.
Data accessibility	Data with this article
Related research article	M.A. Caminha, V.M.B. Lorena, W.O. Júnior, J. Perales, P.C. Carvalho, D.B. Lima, M.G.A.M. Cavalcanti, S.M. Martins, R.H. Valente, R.F.S. Menna-Barreto, *Trypanosoma cruzi* immunoproteome: calpain-like CAP5.5 differentially detected throughout distinct stages of human Chagas disease cardiomyopathy Journal of Proteomics, (2018) pii: S1874–3919(18)30417-2 [1].

**Value of the data**

• The data indicate that the use of DMP for covalent immobilization of human IgG should be averted whenever possible in order to avoid loss of paratope properties;
• For those restricted to DMP usage, the data establish that concentrations of DMP lower than 50 mM should be tested for covalent immobilization of polyclonal antibody samples;
• The data may concern those interested in studying aspects that affect antigen-antibody complex formation;
• These data may be relevant for researchers interested in disabling antigen recognition properties of human IgG.

## 1 Data

**Fig. 1:**
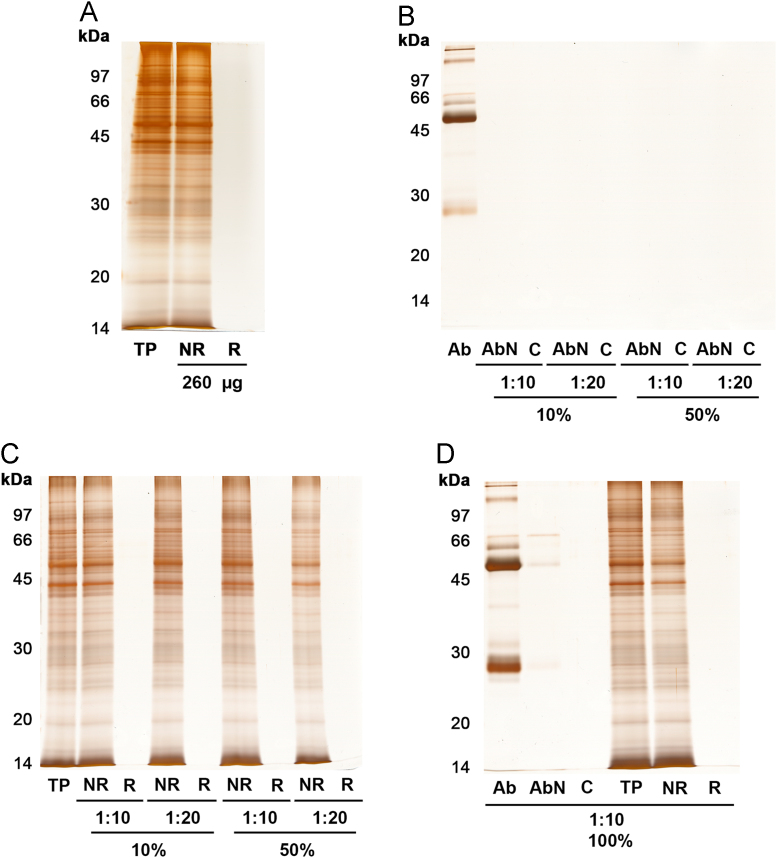
Electrophoretic profile of fractions collected following cross-linking immunoprecipitation assays. (A) Non-specific interaction assay among parasite proteins and magnetic beads; 260 µg of total parasite protein (TP) were loaded onto 2 µL of magnetic beads; (B) and (C) Immunoprecipitation performed with 10 and 50% binding capacity of 2 µL magnetic bead, which corresponded to 2.6 and 13 µg of purified IgG applied in the system, respectively, prior to addition of total parasite protein. Assays were further carried out by loading either 10- (1:10) or 20-times (1:20) of parasite extract in relation to IgG mass previously applied. For each experiment, both (B) IgG immobilization and (C) parasite protein immunoprecipitation efficiencies were monitored by SDS-PAGE; (D) Immunoprecipitation carried out with 100% IgG binding capacity (26 µg) and a 1:10 ratio (m/m) of immobilized IgG to total parasite protein extract. Twelve percent SDS-PAGEs stained by silver impregnation. Ab: original samples of purified IgG; AbN: fractions with eventual not-retained antibody collected at the end of antibody immobilization period (1 h) from each test, which are signalized by the ratio of parasite extract applied afterwards; C: fraction collected immediately after cross-linking reaction; NR: fractions with not-retained parasite proteins; R: fractions containing retained *T. cruzi* proteins after 1 h of incubation at room temperature.

**Fig. 2:**
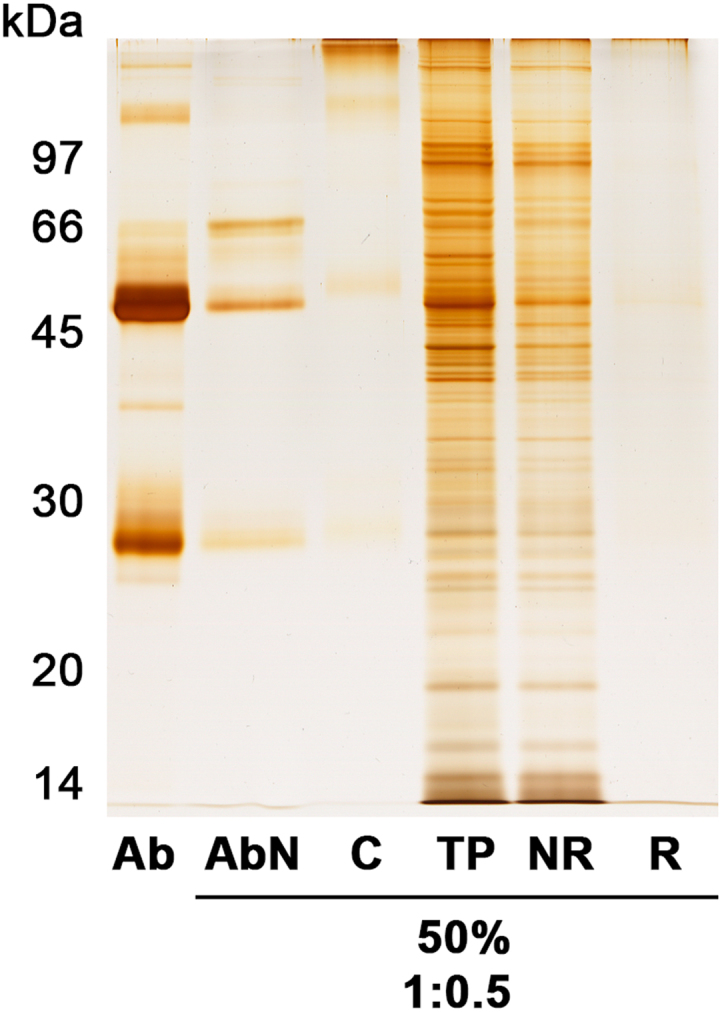
Cross-linking immunoprecipitation assay performed with 50% binding capacity of 10 µL magnetic beads (65 µg of IgG), followed by addition of 37.5 µg of *T. cruzi* extract (ca. 1:0.5 ratio). Ab: original antibody purified sample; AbN: fraction with eventual non-retained antibody after 2 h of incubation for antibody immobilization; C: fraction collected immediately after cross-linking reaction; TP: total parasite protein; NR: fraction containing non-retained parasite proteins; R: fraction with retained proteins after 2 h incubation at 37 °C.

Immunoprecipitation assays were performed by covalent IgG immobilization with 50 mM DMP (dimethyl pimelimidate), which has an imidioester at each end that reacts with amine groups, forming a stable amidine bond [2]. IgG protein quantities equivalent to 10, 50 or 100% of matrix binding capacity (13 µg of IgG/µL of magnetic bead) were tested in combination with different ratios of parasite protein extract. SDS-PAGE analysis of fractions eluted in the cross-linking immunoprecipitation assays did not result in polypeptide chain detection, regardless of growing amounts of either IgG or parasite protein assayed (Fig. 1, Fig. 2).

## 2 Experimental design, materials and methods

### 2.1 Parasite protein sample preparation

Proteins were extracted from bloodstream trypomastigote forms of *T. cruzi* (Y strain) obtained by heart puncture from *Swiss* mice at the 7^th^ day post infection. Parasites were washed thrice with phosphate-buffered saline (PBS, pH 7.4) and centrifuged at 6500*g* for 10 min at 4 °C each time. Proteins were then extracted combining lysis solution (20 mM Tris, 1% Nonidet P-40, 300 mM NaCl, 50 mM KCl pH 7.5, 10 μM E-64) containing CompleteMini protease inhibitor cocktail (Roche Applied Science, Indianapolis, EUA) with 10 cycles of freezing-thawing in liquid nitrogen. Next, fractionation was performed at 16,000*g* for 20 min at 4 °C; the soluble fraction was collected and stored at -80 °C [3]. Protein quantification was carried out with 2-D Quant Kit (GE Healthcare, Buckinghamshire, England).

### 2.2 Human IgG purification

Serum was collected from a Chagas disease carrier with diagnosed cardiomyopathy stage C [4] assisted at the “*Ambulatório de Doença de Chagas e InsuficiênciaCardíaca*”, coordinated by the Prof. Luiz Tavares Cardiologic Emergency Room of Pernambuco (PROCAPE) of the University of Pernambuco, Brazil. Prior to sample collection, the individual was informed about the study and consent. This work followed the ethical principles in human experimentation adopted by the local Research Ethics Committee (CEP/CPqAM: 33/10, CAAE: 0032.0.095.000-10).

IgG purification was performed by affinity chromatography with a Protein G column (HiTrap rProtein G 0.7 ×2.5 cm, GE Healthcare) connected to an Äkta Purifier (GE Healthcare). Sample was diluted 10-fold in 20 mM sodium phosphate pH 7.0 and soluble fraction was obtained after a centrifugation step at 16,000*g* for 10 min at 4 °C. Following an equilibration step with the same buffer, 4 mg of protein were applied onto the column; analyte was eluted with 100 mM glycine-HCl pH 2.1 and its pH was immediately neutralized with 1 M Tris pH 9.0. The chromatographic run was carried out at room temperature, a flow rate of 1 mL/min and absorbance monitoring at 280 nm. Pool of purified IgG was prepared as previously described for the immunoprecipitation experiments [1].

### 2.3 Cross-linking immunoprecipitation assays

The immunoprecipitation experiments were performed with either 10 or 50 µL of ressuspended slurry, which were equivalent to 2 or 10 µL of Protein G magnetic beads (GE Healthcare). Particles were initially equilibrated with TBS (50 mM Tris, 150 mM NaCl, pH 7.5). Next, 100 µL of IgG were applied into the system and maintained at room temperature for 1 or 2 h, respectively; antibodies eventually not bound to Protein G were collected (AbN). After washing the magnetic beads with TBS, 200 mM triethanolamine pH 8.9 were added; beads were homogenized and the solution was rapidly discarded. Following, cross-linking reaction was performed with 50 mM of dimethyl pimelimidate (DMP) (Sigma-Aldrich, Saint Louis, EUA) in triethanolamine for 1 h at room temperature. Beads were then ressuspended with 200 mM triethanolamine pH 8.9. The solution was removed and the reaction was blocked by adding 100 mM ethanolamine pH 8.9. After 15 min, supernatant was removed and the eventually not retained antibodies were eluted with 0.1 M glycine-HCl pH 2.9 added 2 M urea. This fraction was collected and identified as “C”. Beads were then equilibrated twice with TBS and the parasite protein extracts diluted in TBS were added; incubations were carried out at room temperature or 37 °C for 1 h or 2 h, respectively. *T. cruzi* proteins not retained were collected and beads washed thrice with TBS added 2 M urea. Next, immunoreactive proteins were eluted twice with 100 µL of 100 mM glycine-HCl pH 2.9 under moderate shaking by inversion for 10 min; both fractions were pooled. Unless otherwise specified, incubations were performed with 500 µL of solution and incubations were carried out under moderate shaking by inversion.

### 2.4 SDS-PAGE under reducing conditions

Electrophoresis were performed with stacking and separating gels composed by 4% and 12% of acrylamide (30% T, 2.67% C; Biorad, Hercules, USA). Gels were prepared with 0.5 M Tris–HCl pH 6.8 and 1.5 M Tris–HCl pH 8.8, respectively, to a final concentration of 0.1% SDS. Polymerization and runs were performed in Vert-i10 system (Loccus Biotechnology, São Paulo, Brazil). Samples were solubilized in modified Laemmli [5] buffer (0.06 M Tris–HCl pH 6.8, 2% SDS, 100 mM dithiothreitol, 10% glycerol, 0.025% bromophenol blue), boiled for 5 min, centrifuged at 16,000*g* for 2 min and then applied onto the gels. Electrophoreses were performed at constant 200 V in Tris-glycine buffer (25 mM tris, 192 mM glycine, 0.1% SDS pH 8.3). GE Healthcare mixture of standard molecular mass was used as reference. At the end of electrophoresis, gels were stained by silver impregnation. Polypeptide chains fixation were carried out overnight with 30% (v/v) ethanol and 10% (v/v) acetic acid. Next, a sensitization step was performed with 30% (v/v) ethanol, 0.2% (w/v) sodium thiosulfate and 12% (w/v) sodium acetate trihydrate for 30 min. Gels were then washed thrice (2 min each) with deionized water and incubated for 20 min in 0.25% (w/v) silver nitrate and 0.04% (v/v) 37% formaldehyde. Following three washes of 20 s each, polypeptide chains were detected with 2.5% (w/v) sodium carbonate, 0.02% (v/v) formaldehyde, 0.001% (w/v) sodium thiosulfate. The detections were carried out for 2 to 5. Next, this solution was discarded and the gels were maintained for 10 min in 1.5% (w/v) EDTA-Na2.2H_2_O [6]. Gel images were acquired using an ImageScanner III (GE Healthcare) and Adobe Photoshop Elements (Adobe Systems Inc.).
