# Supplementary material for: Data on antigen recognition hindrance by antibodies covalently immobilized to Protein G magnetic beads by dimethyl pimelimidate (DMP) cross-linking
Source: Data Brief. 2018 Dec 21;22:516–21. doi: 10.1016/j.dib.2018.12.057 (PMC6327068; doi:10.1016/j.dib.2018.12.057)
Supplement: Supplementary file 1 — Transparency document [file mmc1.docx]

**Conflict of interest**

None to declare.
